# Applying team-based learning in a transnational post registration bachelor of nursing program in Singapore

**DOI:** 10.1186/s12912-021-00593-4

**Published:** 2021-05-24

**Authors:** Rob Burton, Thea van de Mortel, Victoria Kain

**Affiliations:** School of Nursing and Midwifery, Griffith University, Nathan, Queensland Australia

**Keywords:** Team-based learning, Transnational education, Active learning, Nursing students

## Abstract

**Background:**

Team-Based Learning (TBL) is growing in popularity as a method to create active learning within larger group lectures. It is facilitated through phases of individual work, teamwork with immediate feedback and an application exercise, to develop students’ understanding and assessment of conceptual knowledge. A single facilitator can manage many groups within larger lectures. The study aim was to evaluate the impact of TBL on the engagement, learning and satisfaction of students enrolled in a transnational post registration Bachelor of Nursing (BN) program in Singapore.

**Methods:**

A cross-sectional design was employed. The TBL approach was delivered during lecture sessions within a post registration BN program delivered in a Higher Education Institution in Singapore. A sample of BN students was drawn from 305 students undertaking the program. An online anonymous university-delivered student evaluation of course (SEC) survey and an online anonymous survey using the Student Assessment Instrument, were used to collect quantitative and qualitative data. Survey participation was voluntary with informed consent protocols followed. Student performance in the course was also reviewed.

**Results:**

Eighty-two students (27%) completed the SEC scoring a median of 4/5 for satisfaction, and 68 (22%) completed the online survey. As 93 was the neutral score for the survey, there was a moderately positive evaluation with an overall score of 108.5/155 for TBL in accountability for learning, TBL preference and satisfaction with TBL compared to traditional lecture approaches.

**Conclusions:**

Implementation of TBL with this cohort demonstrated evidence of moderately positive engagement, learning and satisfaction when compared to traditional didactic lectures.

## Background

Many East-Asian students accessing Higher Education experience didactic teaching methods [1]. Some Asian cultures foster an atmosphere of passive learning, respectful deference to the authority of the teacher and/or text, and consequently students become accustomed to situations where they are attentive to the speaker in structured, managed, environments. This can lead to a reluctance to critique or question in group or collaborative situations [2]. However, such students have also demonstrated adaptability to other instructional approaches and can gain deeper learning with appropriate exposure [3, 4]. Team-Based Learning (TBL) is an active learning approach that is growing in popularity and is increasingly utilised as a teaching approach in medical and other health professional education programs [5–7]. As there are no published studies specifically related to TBL in nurse education conducted in Singapore, this project was undertaken to evaluate the application of TBL within a transnational Bachelor of Nursing (BN) post-registration program for Singaporean nurses. In this context, TBL was implemented to replace large-scale didactic lectures previously delivered within the program.

Economic and demographic trends are impacting higher education institutions, and many seek to minimise inefficiencies and maximise revenue by increasing student numbers [8]. These factors inevitably lead to growing class sizes and a reliance on didactic lectures as a method to impart course information to large student numbers [9]. Given these pressures it is imperative to maintain quality approaches to student learning.

A systematic review and meta-analysis of 225 studies involving over 29,000 science, technology, engineering and mathematics students demonstrated that active learning approaches reduced fail rates by 55% and improved grades by up to half a grade band when compared to didactic teaching methods [10]. Despite these findings, a study [11] that surveyed nurse educators found that only around 5% of them did not use lectures at all, and some educators stated that lectures were used by them up to 75% of the time, suggesting that although active learning is utilised and can be seen as beneficial, large-scale lectures are still prevalent.

Studies suggest that TBL is a useful approach to providing active learning within large class settings. For example, a systematic review [12] noted that between 2011 and 2016, there were 87 TBL studies involving health professionals alone; over triple the number from the previous 5 years. Team-Based Learning involves small group instructional approaches facilitated through structured phases of individual work, teamwork and immediate feedback, to develop students’ understanding and assessment of conceptual knowledge [13]. Furthermore, teaching cost reductions can occur due to the structured nature of the approach. A single facilitator can manage many groups within larger lecture type settings, requiring fewer resources and creating advantages over other types of small group active learning methods [14]. Additionally, a study in a medical school in Singapore found an online version of TBL could provide easily accessible data related to the performance of individuals and teams in each stage of TBL, if embedded within the learning management system [15].

Studies have shown favourable outcomes in terms of student results and satisfaction, comparable to, or higher than, other instructional methods. For example, meta-analyses specific to TBL have calculated significant positive effects on academic outcomes. Two meta-analyses that included mostly medical and pharmacy graduates calculated a mean effect size of 0.55 on content knowledge [16] and a nearly 0.5 standard deviation (SD) increase in average academic outcomes [17]. A meta-analysis showed significantly increased standardised mean differences (SMD) in theoretical examination scores and learning skills for various medical school subjects in China [18]. Similarly, a study [19] was conducted using a pre/post-test design to compare grade outcomes of a lecture-based course conducted over a regular semester with the same course taught over a summer semester using TBL, with nursing students in the United States of America (USA). Academic outcomes were significantly better by nearly 7% in the TBL course when assessed using a national standardised examination. Two systematic reviews have also reported moderate positive effects on academic outcomes, particularly for lower achieving students [12; 20]. TBL outcomes with mostly medical and pharmacy students in the USA were reviewed in one [12], whilst the other study [20] focused almost exclusively on undergraduate nursing and midwifery courses, mostly from the USA. A further study [21] also had similar findings regarding TBL outcomes.

Attitudes and satisfaction levels were also examined in four of the reviews and one meta-analysis. A significant increase was found in Chinese medical students’ positive attitudes towards learning using TBL [18]. Another systematic review also reported overall higher student participation and enjoyment in TBL courses compared with standard lectures, albeit with some reluctance towards the change from lectures to TBL [20]. These findings are comparable to another study which showed attitudes toward TBL tended to improve with time [12]. This review also examined faculty attitudes, concluding that despite the additional workload associated with TBL, faculty approved of the increase in student engagement, and believed that the benefits of TBL outweighed the additional workload.

Overall, these reports suggest that TBL has positive effects upon academic outcomes and indicate moderate satisfaction and preference for TBL over other teaching methods. Positive academic outcomes and TBL attitudes have also been demonstrated specifically for nursing courses. However, the literature specifically regarding the outcomes of the TBL approach on the engagement, learning and satisfaction of nursing students is limited, even more so for East-Asian nursing students. While TBL has been adopted in some institutions in Singapore in areas such as medicine, and engineering, there are no published studies addressing its use in undergraduate nursing education in Singapore.

Within a transnational education BN program delivered in Singapore, a model of keynote lectures followed by tutorials was the approach previously employed. However, as class sizes were growing (to over 300) and more growth in enrolments was planned, a different approach was needed to lecture delivery to ensure more active learning could take place and to increase student satisfaction. Team-Based Learning was introduced as a pedagogical method utilised in the timetabled lecture sessions. Given the absence of literature on the outcomes of TBL in Singaporean nursing students, the research aim was to evaluate the implementation of this model in this cohort.

The research question was: What is the impact of TBL on student engagement, accountability for learning, self-reported preference for learning, and satisfaction in a transnational course for post-registration Singapore nursing students? We also examined the aspects of TBL that students found helpful for their learning, what the challenges were, and report on aspects of applying TBL with this cohort.

## Methods

### Design

A cross-sectional survey design was used to collect quantitative and qualitative data to address the research question. This included the ‘Student Evaluation of Course’ (SEC) and an additional online survey. Grade outcome data were also collected from this course and other courses completed in the previous 18-month period.

### Setting and sample

The study was conducted within an on-campus delivered ‘Professional Communication for Nurses’ course in a Bachelor of Nursing post-registration program delivered to a cohort of 305 Singaporean registered nurses in Singapore between February and July 2018.

### TBL delivery

Four timetabled TBL sessions of four hours duration each were delivered across two teaching blocks in one trimester. There were also some computer lab sessions and tutorials where the students could access the materials and content to be covered in the preparation phase. As this was students’ first experience of TBL, the TBL approach was introduced to them during their first timetabled computer lab and they were provided with live training on the process in the first lecture. Team-Based Learning was delivered in structured phases of preparation, readiness and application. All materials were made available to students for self-directed study for the preparation phase. Tutorials delivered by tutors appraised of TBL processes were used to support students focussing on the course content. The formal TBL approach of an individual readiness assurance test (iRAT), a team readiness assurance test (tRAT) and a team application (tAPP) exercise [20] was then implemented in the subsequent large group lectures in the course. The stages followed can be seen in more detail below [22]:

#### Advance assignment (outside of class)

Students were directed to the course/task outcomes and the resources needed to prepare them for this and were pre-assigned to teams. These materials were accessible via the Virtual Learning Environment (VLE) and discussed in tutorials. Students undertook this preparation both independently and within timetabled computer lab sessions.

#### Individual readiness assurance test (iRAT)

Students undertook a multiple-choice iRAT in the lecture setting. There were 50 questions and students were required to answer within fifty minutes. The students completed the test and recorded their answers in readiness for discussion within the team element. At this stage answers were not revealed.

#### Team readiness assurance test (tRAT)

The teams discussed the same questions covered in the iRAT in class and provided a team response to these. This took fifty minutes. Subsequently, the answers to each question from each team were displayed on a large card indicating A, B, C, D and shared with the larger class, with correct answers still not revealed. This took around twenty-five minutes and gave the facilitator an indication of the similarities and differences in team answers, and therefore also which questions were more difficult or easy for the group as a whole. Following this a scratch card was used (Immediate Feedback Assessment Technique; IF-AT), giving each team an opportunity to redo the tRAT and three opportunities to get the answer correct. The iRAT provides a single point per question and the tRAT provides a maximum of three and a minimum of zero per question, depending on how many attempts the team takes for each question. A correct answer first time in the tRAT would give a score of three, two for a second attempt, one for a third attempt and zero if all four options were accessed. This took around 30 min. The teams could then record their overall score up to a total of 150/150. Individuals could also identify their own score from the iRAT at one point each and score up to 50/50. The IFAT provides immediate feedback to the students, which has been linked to positive outcomes and student satisfaction in some studies [23]**.** The answers are immediately apparent as the scratch card has an asterix under the correct answer. Individuals can then ascertain their own score as well as the team score. Students and teams were then required to hand in the response sheets.

#### Instructor clarification review

Following revelation of the correct answers, the teacher discussed the issues identified in the tests and addressed any queries raised. Any questions that had been problematic for the students were identified and answers clarified. Here the teacher referred to the material that the students had been exposed to in the VLE or by other sources and provided brief explanations for clarification. At this stage students were also allowed to challenge, appeal or debate if they felt the answer was not correct, provided they gave a rationale with supporting evidence. This took 30 to 40 min.

#### Team application (tAPP)

Following the iRAT and the tRAT the students were provided with a problem/ scenario they must solve in a 45-min period. They provided predictions and solutions to the problem and reported back on this via a shared online presentation platform. An example scenario is: *A female patient from another country has been admitted to your ward. English is not the patient’s first language. Drawing on what you have learnt and the use of the SOLAR/SOLER strategies, how would you seek to address the communication issue between yourself and the patient?*

#### Peer review/evaluation

When the three stages were finished the students underwent peer review using a scoring mechanism based on a ‘thermometer scale’ (cold [Passive, not contributing], warm [active, contributing], hot [controlling, confrontative]) and provided qualitative constructive comments on their team members’ teamwork.

### Recruitment and data collection

Before recruiting participants, ethics approval was obtained following a university ‘Human Ethics Review’ of the proposal and all processes were conducted in accordance with relevant regulations, policies, guidelines and ethical procedures. Participants were recruited via an email sent to all enrolled students to which the information sheet and consent form were attached along with a link to the anonymous online survey. Participation was voluntary and anonymous, therefore completing the survey was acknowledgement of consent. No personal data were collected.

The survey instruments included the *Student Experience of Course (SEC),* which is the standard anonymous university course assessment instrument, and a separate online anonymous *TBL survey* based on Mennenga’s validated *TBL-SAI* (student assessment instrument) to investigate TBL experience and attitudes [24]. The SEC has five fixed response questions on a 5-point Likert scale examining student satisfaction with various aspects of the course such as teaching, assessment and delivery. The expectation is that each course obtains a minimum mean score of 3.5 in the SEC. Qualitative data were also collected from this tool via open-ended questions seeking comments on what students found particularly good about the course and how the course could be improved.

The author of the TBL-SAI, Associate Professor Heidi Mennenga [24], gave approval for its use in this study. The SAI instrument was slightly modified to align with the population/sample involved by the removal of two items that did not apply to the TBL approach taken with the participating students. Additional questions were provided for further ratings on the TBL process. Qualitative comments were also collected related to the student’s experiences, adjustment, the stages of TBL and enjoyment, their favourite and least favourite aspects, most and least clear aspects, and suggestions for improvement to the process. The survey took no longer than 20 min to complete. The 31-item scale TBL-SAI (originally 33 in the unmodified version) used a five-point Likert scale to assess TBL experience by assessing three main domains: student accountability, preference for TBL over traditional lectures, and TBL satisfaction. Possible total subscale score ranges are 6–30 (18 neutral), 16–80 (48 neutral), and 9–45 (27 neutral) for accountability, preference, and satisfaction, respectively. Scores lower than the neutral indicate negative attitudes towards the domains. Higher scores indicate higher accountability for learning, a preference for TBL over lectures, and satisfaction with TBL. Overall scores are calculated via the addition of the subscale scores, and thus range from 31 to 155 (93 neutral). Higher scores are positively correlated with a positive TBL experience. As well as being validated by the author of the SAI, the tool has been further validated in other studies [25, 26] that found it consistent and reliable in determining students’ preferences with the TBL.

The SEC survey was distributed by the university in the online VLE platform. The TBL-SAI survey was hosted on ‘Survey Monkey’. The online survey was not released until after the students had completed the course, assessments and the SEC to reduce any impact/influence that the lecturer may have had on students’ opinions, or motivation to complete the survey. No demographic data were requested as there was no intention to compare data amongst different age or gender groups in the analysis. The only criterion was that all participants were registered nurses in Singapore undertaking the course as part of the post-registration BN program.

### Data analysis

Analysis of the quantitative data from the online survey was performed via descriptive statistical analyses using SPPS Version 24. Scale reliability was measured using Cronbach’s alpha. Student assessment results and the SEC data were compared with previous performance in this program.

Qualitative comments from the SEC and from the qualitative aspect of the online survey were placed into NVivo 12 and analysed via thematic analysis [27] to identify and describe the key aspects of the student TBL experience and opinion. Thematic analysis requires a logical, traceable and clearly documented process to be trustworthy [28], so the following six steps were followed by the team:

1. *Data familiarisation* - reading and re-reading the qualitative comments derived from the surveys so that initial ideas can be recorded. This was conducted by the main researcher.
2. *Data coding* **–** assigning codes to significant aspects of the data in order to group similar data together. The comments were placed in NVivo 12 and codes applied to each one where a clear concept was expressed.
3. *Theme search* – collating the coded data into potential themes. The coded comments were reorganised into structured themes containing similar coded content.
4. *Theme revision* – revising the identified themes in order to create a thematic ‘map’ that relates to both the codes and the entire data set. The researcher utilised NVivo to produce an infographic visualising the formation of the themes.
5. *Theme definition* – naming and clearly defining each theme. Each theme was reduced by the researcher until the final themes were determined and these were shared with the wider team for review.
6. *Writing up* – reporting the analysis with relevant code and theme examples and providing a thematic ‘map’ [27].

## Results

A population of 305 students were eligible to participate. Eighty-two students (27%) completed the SEC and 68 (22%) completed the online survey.

### Online survey

Table 1 displays the means and standard deviations (SD) for the TBL Student Assessment Instrument. These results show moderately positive outcomes for all three subscales, resulting in a moderately favourable overall experience with TBL compared to lectures. Scale reliability was satisfactory for the accountability (.737) and TBL preferences (.774) scales, and excellent for the TBL satisfaction scale (.921).

**Table 1:** The Team-Based Learning Student Assessment Instrument descriptive statistics

*Subscale*	*N*	*Maximum possible score*	*Mean (SD)*
Accountability	68	30	22.2 (2.8)
Preference	68	80	52.7 (6.3)
Satisfaction	67	45	33.5 (5.1)
Overall	67^a^	155	108.5 (12.2)

^a^One participant did not answer the questions relating to the satisfaction subscale and thus could not be included

Table 2 displays the means and standard deviations for the remaining questions. These were rated on a scale from 1 to 10. Higher scores indicated students favoured TBL. The results show modest positive results for all questions.

**Table 2:** Descriptive statistics for the additional questions

*Question (descriptive answer range)*		*N*	*Mean (SD)*
How would you rate your experiences of the Team Based Learning approach overall? (poor to excellent)		68	6.8 (1.6)
How difficult/easy was it to adjust to learning via the TBL process? (very difficult to very easy)		67	6.2 (1.6)
How would you rate …? (poor to excellent)	… the engagement or interaction experienced in TBL classes?	68	6.7 (1.5)
	… your own level of learning from the TBL process?	68	6.6 (1.3)
Please rate the following TBL steps in terms of how useful each was to your learning. (not useful to highly useful)	The (self) preparation readiness assurance phase	69	6.3 (1.7)
	The individual (iRAT) and team test (tRAT) processes	70	6.4 (1.5)
	The IF-AT test resources (Scratch card process)	69	6.5 (1.7)
How enjoyable were the individual (iRAT) and team test (tRAT) processes? (not at all to very enjoyable)		70	6.8 (1.7)

### Student evaluation of course (SEC)

The SEC asks student to agree or disagree with the following statements on a 5-point Likert scale: Q1 ‘This course was well-organised’; Q2 ‘The assessment was clear and fair’; Q3 ‘I received helpful feedback on my assessment work’; Q4 ‘This course engaged me in learning’; Q5 ‘The teaching (lecturers, tutors, online etc) on this course was effective in helping me to learn’; Q6 ‘Overall, I am satisfied with the quality of this course’. These scored a median 4/5 on the Likert scale in each item.

### Performance

Table 3 displays the grade distribution achieved by the whole cohort in the course delivered using TBL. The overall mean score in this course was 72.89% (SD = 12.63) compared to a mean of 63.96% (SD 2.42) across the other four courses delivered in the program during the previous 18 months.

**Table 3:** Students’ course score performance comparisons across the programme

Comparison of course mean scores	Course 1	Course 2	Course 3	Course 4	Overall Mean	SD
Other Course mean scores	59.84	64.7	66.01	65.32	63.9675	2.43
TBL Course					72.89	12.63

### Qualitative data

Qualitative data were obtained from the SEC qualitative statements, and from the qualitative element of the online survey tool. These were analysed via NVivo 12.

**Fig. 1:**
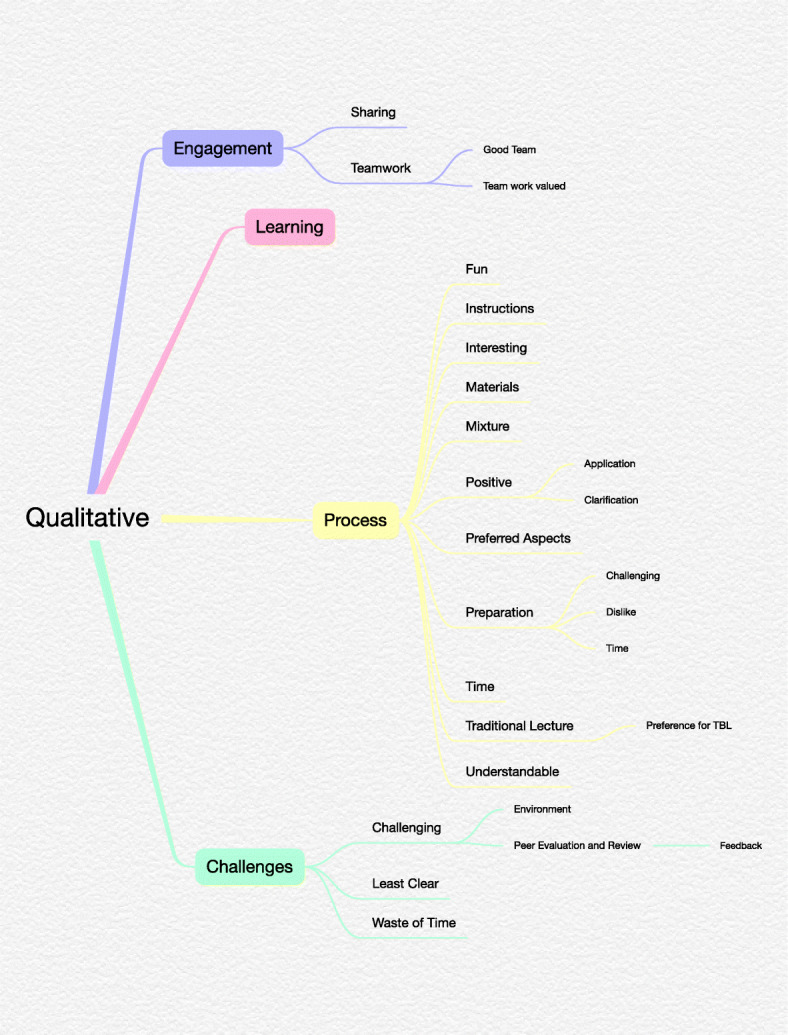
Thematic Analysis

Four themes were developed following 245 original code references, and 17 theme revisions highlighted within the qualitative texts. These themes were *Engagement, Learning, Process*, and *Challenges*. Overall, the responses were positive about the experience of TBL, although some students did express some concern about the approach. This can be seen in Fig. 1 below:

#### Theme 1: engagement

This theme drawn from 40 reference statements, encapsulated aspects related to activity, engaging and sharing with others, and team working as a result of the TBL process. Students appeared to enjoy the opportunity for working in teams and interacting with others:

*‘Team based learning approach was very engaging and beneficial in understanding the course better.’*

*‘Team based learning used in teaching is a good approach. It engages students more than traditional teaching methods.’*

#### Theme 2: learning

This theme was drawn from 24 reference statements. These were related to the students demonstrating positive attitudes towards the learning experience and the learning/understanding gained in the process. Participants highlighted the value of the TBL approach in relation to their learning:

*‘Team based learning is good to aid in learning process.’*

*‘It’s basically putting my knowledge to a test to see what I have learned has retained.’ (*SIC*).*

*‘Especially at the end of IF-AT & tRAT where we get to challenge the lecturer with our own answers. This promotes critical thinking and allows students to learn in a unique way.’*

#### Theme 3: process

This theme was drawn from 47 reference statements. It refers to the TBL aspects that the students experienced and their perceptions of them. The students seemed to enjoy the team-based learning process:

*‘It is a fun and engaging way of learning and it has certainly benefitted me in my learning journey.’*

*‘Team-based learning is really effective, as followed by feedback session where students can clarify the answers with the lecturer for any doubt or confusion.’*

#### Theme 4: challenges

This theme was drawn from 43 reference statements and relates to aspects students reported as challenging or negative from their perspective. There were some aspects that students found impacted on their engagement:

*‘I recalled the first trial was quite difficult because sometimes we hardly to express our opinions with others whom we don’t know, and we weren’t sure about the flow of TBL and how it worked.’ (*SIC*).*

*‘The members who are non-enthusiastic in contributing to the team’s efforts.’*

Some challenges were identified in relation to learning:

*‘Did MCQ for whole class without explanation, I didn’t feel I’m learning.’*

The process was also not without its challenges:

*‘It is challenging and mentally draining to work on 100 MCQ and 1-2 scenario(s).’*

*‘I prefer combination of traditional lecture with team-based’.*

Environmental factors such as classroom layout were noted as the factors most affecting the approach including the noise when groups were working and discussing in their teams.

*‘Round table will be the best, sitting in a row can be difficult to do discussion’.*

*‘TBL can be a bit noisy. Hard to focus.’*

The students also found the peer review aspect to be challenging as some were reluctant to criticise their colleagues:

*‘No peer evaluations, it’s not true evaluation due to the Asian culture. Nobody will write honestly; majority are positive feedbacks.’ (*SIC*).*

However, some students recognised the value of the peer review:

*‘I like it. If they just say you’re not good or you’re good it doesn’t help, but if it’s constructive feedback, they tell you your improvement area it really helps.’*

## Discussion

The aim of this study was to evaluate the impact of TBL on student engagement, learning, accountability for learning and satisfaction in a transnational post-registration BN course for Singaporean nurses. The quantitative survey data from the TBL SAI demonstrated a moderately positive evaluation of these factors. The quantitative SEC scores also demonstrated overall student satisfaction with the course. Performance outcomes in the course were better when compared to other courses in the program. This finding is in keeping with a systematic review in 2021, which shows that most TBL studies show increased performance outcomes, although some were not conclusive about the effect of TBL compared to traditional methods [29].

The results from the qualitative data collected via the SEC and TBL online surveys demonstrated that students enjoyed the engagement aspect of TBL, which is borne out in comments made, although some comments from the data suggested that the formation of teams was a challenging aspect. This is a necessary process however, and one with several benefits once students work together in their teams as a previous study demonstrated [30]. Another meta-analysis [31], showed significantly improved team scores compared to individual scores, and noticeably so amongst nursing students. Therefore, the issue of team formation is one that needs careful consideration in improving performance. This was also reported as a factor in a study with pharmacy students using the SAI [32] which yielded similar results to this study.

The results from the TBL survey are very similar to those from a study [33] with nursing students in the United Kingdom (UK), that found students also demonstrated positive scores in the satisfaction, accountability and preference scales of the TBL-SAI, indicating a preference for TBL over traditional lectures. Western approaches to learning and teaching might not always be preferred by East-Asian students, who would tend not to speak up when older adults are present in a group, nor would they challenge or question a teacher, however TBL provides them a legitimate arena in which to do both [3]. A previous systematic review [20] also identified positive aspects from using a TBL approach whilst acknowledging that there are still challenges to be overcome, for example in relation to team formation and peer review/evaluation, as was evident in this study.

Peer review was unpopular as students commented that they did not enjoy giving low scores or negative comments. This could be related to the Asian cultural context, which is based on maintaining harmonious relationships, virtuous behaviours and recognising hierarchical relationships [3]. Our findings in relation to peer review differ from a study [34], which found that the peer review process increased students’ accountability in team tasks as they consistently found that team members that were not engaged tended to receive low scores and evaluations, which the participants in our study were reluctant to do. The results from this study in relation to student satisfaction were also similar to a study which found positive effects, yet also mentions the challenges faced in peer review or assessment [35].

In relation to learning the results were moderately positive, both in terms of self-reported learning on the SAI learning subscale, qualitative comments on both the SAI and SEC surveys, and also in terms of mean percentage scores for the course. Student performance was 9% better in this course when compared to mean scores in other courses delivered in the program over the previous 2 years, therefore almost a full grade higher. These are not ‘like for like’ courses, nor had there been a previous iteration of this course to draw a comparison with, so although outcomes are positive, caution must be applied in attributing this to TBL as other confounding factors may be present. However, a previous study [36], also showed an increase in grade performance after implementing TBL.

In terms of satisfaction, the participants had moderately positive satisfaction with the TBL process, particularly in relation to engagement. Students particularly enjoyed sharing their team answers and the reveal of the correct answers during the IF-AT aspect of the process. The SEC results demonstrated a level of satisfaction equal to or slightly higher than other courses delivered in the same program in the previous 2 years. There were many student comments about how they preferred TBL to traditional lectures as they were actively involved, and it ‘wasn’t boring’. A study [37], also found that Korean nursing students were generally satisfied with TBL, suggesting it has potential as a pedagogical approach with Asian students. However, some students suggested they would still like some formal traditional lectures as well as the TBL.

### Limitations

The survey response rates were low (22–27%), and we did not collect demographic data, limiting the ability to generalise these findings and attribute causation to the TBL approach, however, online surveys tend to have response rates (average 34% across 207 studies) up to 15% lower than paper-based surveys [38]. The closeness of the lecturer to the delivery of the TBL and the research process is also a limiting factor, although the surveys were anonymous and the data were collected after grades had been finalised, which should reduce any potential related bias. While it is possible that there was a selection bias towards those students who had a positive experience with TBL there were still comments that some students preferred traditional lectures.

## Conclusions

Implementation of TBL with this East-Asian cohort on a transnational program demonstrated evidence of moderate positive engagement, learning and satisfaction scores when compared to traditional didactic lectures. Most students enjoyed TBL and preferred it to their previous experiences of didactic lectures. The peer review process was challenging and not enjoyed by students, as they did not wish to appear critical of their colleagues, nor be humiliated within their team. A more anonymous online version of peer review may help students be more forthcoming in this area. In order to implement TBL and include it as a major pedagogy in nursing programs, particularly in an East-Asian cohort, care is needed in preparing the students, particularly in relation to team formation and addressing issues with reluctance to perform peer review. Grade outcomes, which were higher compared to other courses in the program, provide some level of evidence that TBL can positively impact learning and performance of East-Asian nursing students.

Further research is required in this setting to quantify learning outcomes from utilising a TBL approach and to make wider comparisons with other demographic groups. More studies evaluating comparisons of the TBL approach in face to face or online situations would also be useful given the pivot towards online teaching driven by the recent pandemic.

## Data Availability

The datasets used and/or analysed during the current study are available from the corresponding author on reasonable request.

## Abbreviations

BN: Bachelor of Nursing
IF-AT: Immediate Feedback Assessment Technique
iRAT: Individual Readiness Assurance test
SAI: Student assessment instrument
SD: Standard deviation
SEC: Student evaluation of course
SMD: Standardised mean differences
tAPP: Team Application
TBL: Team Based Learning
tRAT: Team Readiness Assurance Test
USA: United States of America
VLE: Virtual Learning Environment
